# Prediction of Aptamer-Target Interacting Pairs with Pseudo-Amino Acid Composition

**DOI:** 10.1371/journal.pone.0086729

**Published:** 2014-01-22

**Authors:** Bi-Qing Li, Yu-Chao Zhang, Guo-Hua Huang, Wei-Ren Cui, Ning Zhang, Yu-Dong Cai

**Affiliations:** 1 Institute of Systems Biology, Shanghai University, Shanghai, P.R. China; 2 Key Laboratory of Systems Biology, Shanghai Institutes for Biological Sciences, Chinese Academy of Sciences, Shanghai, P. R. China; 3 State Key Laboratory of Medical Genomics, Institute of Health Sciences, Shanghai Jiaotong University School of Medicine and Shanghai Institutes for Biological Sciences, Chinese Academy of Sciences, Shanghai, P. R. China; 4 CAS-MPG Partner Institute for Computational Biology, Shanghai Institutes for Biological Sciences, Chinese Academy of Sciences, Shanghai, P. R. China; 5 Department of Biomedical Engineering Tianjin University, Tianjin Key Lab of BME Measurement, Tianjin, P. R. China; University of Rome, Italy

## Abstract

Aptamers are oligonucleic acid or peptide molecules that bind to specific target molecules. As a novel and powerful class of ligands, aptamers are thought to have excellent potential for applications in the fields of biosensing, diagnostics and therapeutics. In this study, a new method for predicting aptamer-target interacting pairs was proposed by integrating features derived from both aptamers and their targets. Features of nucleotide composition and traditional amino acid composition as well as pseudo amino acid were utilized to represent aptamers and targets, respectively. The predictor was constructed based on Random Forest and the optimal features were selected by using the maximum relevance minimum redundancy (mRMR) method and the incremental feature selection (IFS) method. As a result, 81.34% *accuracy* and 0.4612 *MCC* were obtained for the training dataset, and 77.41% *accuracy* and 0.3717 *MCC* were achieved for the testing dataset. An optimal feature set of 220 features were selected, which were considered as the ones that contributed significantly to the interacting aptamer-target pair predictions. Analysis of the optimal feature set indicated several important factors in determining aptamer-target interactions. It is anticipated that our prediction method may become a useful tool for identifying aptamer-target pairs and the features selected and analyzed in this study may provide useful insights into the mechanism of interactions between aptamers and targets.

## Introduction

Aptamers, first identified by three laboratories independently in 1990 [1], [2], [3], are synthetic single-stranded nucleic acids or peptides. These artificial molecules folding into specific spatial conformations can bind to certain targets with extremely high specificity. They mimic properties of antibodies, but possess several advantages compared with antibodies. Firstly, aptamers can probably target any molecules across the range from small inorganic ions to intact cells, since they are synthesized and selected *in vitro* based on affinities for recognizing their objective targets. Secondly, after selected, aptamers can be easily amplified through polymerase chain reactions to obtain large quantities of high-purity molecules. Finally, aptamers with simple chemical structures can be easily amended by adding some functional groups making the molecules more stable than antibodies in harsh conditions. Thus aptamers, as a novel and powerful class of ligands, are thought to have excellent potential for applications in the fields of diagnostics, therapeutics and biosensing [4], [5].

Typical approach of selecting aptamers is systematic evolution of ligands by exponential enrichment (SELEX) [1], [2] initiating with a stochastic library containing single-stranded DNA or RNA sequences. This conventional method for generating aptamers *in vitro* or *in vivo* [6], [7], [8], [9], [10] from random combinatorial libraries is often labor-intensive and time-consuming, taking weeks to months to finish. Although a plenty of efforts have been put forward to improve the aptamer selection, it is still desirable to develop a computational method for designing effective aptamers binding to certain interested targets, saving much time and labor.

In this study, a new method for predicting aptamer-target interacting pairs was proposed by integrating information from both aptamers and their targets. Each aptamer was represented with 20 features by nucleotide composition. And each target protein was encoded with another 270 features, by using amino acid composition and pseudo-amino acid composition containing electrostatic charge, codon diversity, molecular volume, polarity, and secondary structure. Subsequently, the Maximum Relevance Minimum Redundancy method (mRMR) and the Incremental Feature Selection (IFS) method were adopted to select the optimal features for the prediction. Result might provide strong implications in developments and improvements to broaden the applications of aptamers in biochemical and medicinal fields.

## Materials and Methods

### Dataset

Apatmer Base (http://aptamer.freebase.com/) is a collaborative knowledge base about aptamers, containing their interactions and detailed experimental conditions with citations to primary scientific literature [11]. It contains a total number of 1638 entries of interactions (accessed in Sep. 2012), 1381 of which are aptamers of DNA or RNA interacting with 211 target proteins. Since only protein names of target proteins are provided in Apatmer Base, such as human interleukin 17A, prothrombin, Human toll-like receptor 3 ectodomain, etc., we searched Swissprot for their sequences according to the best name matches. Only 168 protein sequences can be found with exact name matches. And we removed 4 proteins since their lengths were less than 50 amino acids. Finally, 164 proteins with sequences were obtained as apatmer targets corresponding to 1554 interactions between apatmers and proteins.

In the 1381 aptamers, only 725 interact with the 164 proteins. Thus the 725 aptamer-target pairs are regarded as positive samples. 2175 negative samples were generated by randomly pairing the aptamers and the protein targets, with no overlap with the positive samples. The dataset was randomly split into two parts, one for training containing 580 positive and 1740 negative samples and the other for testing containing 145 positive and 435 negative samples. The dataset was given in Additional File S1.

### Feature Construction

In this study, nucleotide composition was employed to encode the aptamer sequences. And amino acid composition and pseudo-amino acid composition were adopted to encode the target protein sequences.

### Nucleotide Composition

Since U in RNA is similar to T in DNA. Therefore, we encoded them in the same way. Then the compositions of A, T/U, C, G, AA, AC, AT/AU, AG, CA, CC, CT/CU, CG, TA/UA, TC/UC, TT/UU, TG/UG, GA, GC, GT/GU and GG were computed to encode each aptamer. Thus each aptamer was encoded into a 20-dimensional numerical vector.

### Amino Acid Composition

Amino acid composition is a type of basic feature of protein sequence, which is closely related to many protein attributes, such as subcellular location [12], [13], [14], domain [15], folding type [16] and secondary structure [17]. Amino acid composition includes 20 discrete numbers, each of which represents occurrence frequency of each of the 20 native amino acid in a protein sequence, respectively. In this study, each protein was encoded into a 20-dimensional numerical vector by using the amino acid composition.

### Pseudo-Amino Acid Composition

The concept of pseudo-amino acid composition (PseAAC) was first proposed by Chou for predicting protein cellular attributes [18]. Based on the conventional amino acid composition, Chou proposed a set of discrete numbers to consider possible sequence order patterns. PseAAC has been proved to be a type of effective features in many biological problems [19], [20], [21]. The concept of PseAAC can be described as follows.

Suppose a protein sequence of *L* amino acid residues:
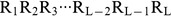
(1)


The sequence order effect of the protein can be represented by a set of discrete correlation factors, which are calculated as follows:
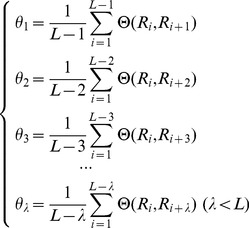
(2)where 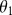, 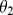, 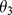, 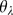 are the first-tier, second-tier, third-tier, 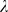 *-th* tier correlation factors, respectively. And the correlation function is
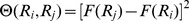
(3)where 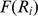 is the feature (e.g. electrostatic charge) value of the amino acid 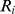. The value is converted from the original feature value of the amino acid according to the following equation:
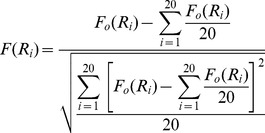
(4)where 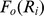 is the original feature value of the amino acid 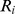. Then the PseAAC of a protein can be represented by a (20+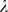)-*D* vector as follows:
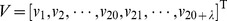
(5)where the superscript *T* is the transpose operator
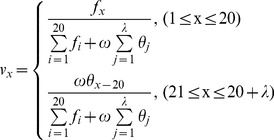
(6)where 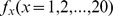 represents the occurrence frequencies of the 20 amino acids in the protein sequence, 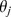 represents the *j-th* tier sequence correlation factor calculated according to Eq. (2), and 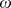 represents the weight for the sequence order effect. Based on the above description, it is known that the first 20 components in Eq. (5) represent the effect of the conventional amino acid composition, while the remaining 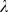 components are the correlation factors representing the effect of sequence order. A set of such 20+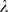 numbers is named PseAAC. In this study, we set 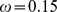, 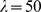.

In this study, polarity, codon diversity, electrostatic charge, molecular volume and secondary structure are used to describe the physicochemical and biochemical properties of amino acids. And the 5 features were retrieved from [22], [23], which can be found in Additional File S2.

### Feature Space

In this study, the nucleotide composition (20-*D*) was used to encode aptamers. The conventional amino acid composition (20-*D*) and the sequence order effect described by the components from 21 to 20+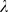 in the Eq. (5) (50-*D*) were adopted to encode targets. Therefore, the feature space is (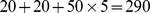)-*D*. In other words, one aptamer-target pair can be encoded into a 290-*D* dimensional vector by the nucleotide composition, amino acid composition and pseudo-amino acid composition containing the codon diversity, electrostatic charge, molecular volume, polarity and secondary structure of amino acids (see Additional file S3).

### Modeling

We first ranked the 290 features by using the Maximum Relevance, Minimum Redundancy (mRMR) method. Based on the ranked feature list, the Incremental Feature Selection (IFS) method was employed to select the optimal feature subset. The prediction model was constructed based on Random Forest and was evaluated by 10-fold cross validation.

### mRMR Method

In this study, the Maximum Relevance Minimum Redundancy [24] (mRMR) method was employed to rank the importance of the 290 features in descending order. The main ideas of the method are that the to-be-selected feature should have the maximum correlation to the target class and should have minimum redundancy to the already selected features. Features are selected from the 290-*D* features one by one and ranked into a MaxRel feature list according to the Maximum Relevance criterion, and also into an mRMR feature list according to both maximum correlation and minimum redundancy criteria. Both the relevance and redundancy are quantified by mutual information (MI) which is defined by
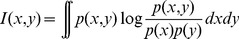
(7)where 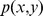 is the joint probabilistic density for feature *x* and feature *y*, 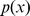 and 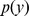 are the marginal probabilistic densities for feature *x* and feature *y*, respectively.

Suppose the whole feature set was denoted by *Ω*, the already selected feature set having *m* features was represented by *Ω_s_* and the feature set with *n* features was denoted by *Ω_t_*, the relevance *D* between the feature *f* in *Ω_t_* and the class *c* is calculated by
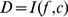
(8)


And the redundancy *R* of *f* with all features in *Ω_s_* is calculated by
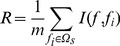
(9)


To select the feature *f*_i_ in *Ω_t_* with maximum relevance to the class and minimum redundancy to the already selected features in *Ω_s_*, Eq. (8) and Eq. (9) are combined together:
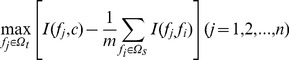
(10)Then the feature *f*_i_ will be removed from *Ω_t_* and be added into *Ω_s_*_._ This process will be repeated until all features are removed from *Ω_t_* and added into *Ω_s_*_._ The better the feature is, the earlier it is selected.

### Random Forest

The Random forest (RF) approach is a popular machine-learning algorithm that has been recently successfully used in dealing with various biological prediction problems [25], [26], [27], [28], [29]. Developed by Loe Breiman [30], RF is an ensemble predictor consisting of multiple decision trees. A queried sample with an input vector will be given a classification by each decision tree in the forest. The forest will choose the class as the final classification that most decision trees in the forest voted. Each tree is constructed according to the following procedure:

1. Suppose the number of training cases is *N*, sample *N* cases at random, but with replacement, from the original data, which will be the training set for growing the tree.
2. If there are *M* input variables, at each node, *m* variables are selected randomly out of the *M* input variables, where *m* is much less than *M*. The most optimized split on these *m* variables is employed to split the node. The value of *m* does not change during the growth of the forest.
3. Each tree is fully grown and not pruned.

In this study, we employed Random Forest implemented in Weka 3.6.4 [31] with default parameters.

### Ten-fold Cross-Validation Method

Ten-fold cross-validation was often used to evaluate the performance of a classifier [32]. During the procedure, the dataset is randomly and evenly split into ten folds, out of which nine folds are used for training and the remaining one for testing. This procedure is repeated ten times and each sample is tested exactly once. To evaluate the performance of the predictor, the prediction *accuracy*, *specificity*, *sensitivity* and *MCC* (Matthews correlation coefficient) were calculated as below:
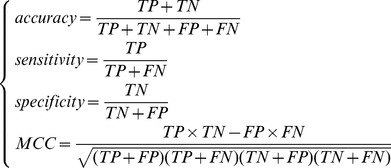
(11)where *TP*, *TN*, *FP*, *FN* denote true positive, true negative, false positive and false negative, respectively.

### Incremental Feature Selection

From the ranked features by mRMR, we used the Incremental Feature Selection (IFS) method [26], [33], [34], [35] to determine the optimal feature set. During the IFS procedure, features in the ranked feature list are added one by one from higher to lower rank. A new feature set is constructed when one new feature is added. Totally 290 feature sets are generated since the total number of features is 290. The i-th feature set is:
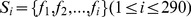
(12)For each feature set, a random forest is constructed and tested by using ten-fold cross-validation. We obtained totally 290 feature sets and correspondingly built 290 random forest predictors. The prediction performances of the 290 predictors were reported in an IFS table, containing the prediction *accuracies*, *sensitivities*, *specificities* and *MCC*s of the predictors. Finally the optimal feature set (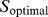) was obtained from the table when the corresponding predictor yielded the best performance.

## Results and Discussion

### The mRMR Result

After running the mRMR software, we obtained two tables (see Additional File S4): one was called MaxRel feature table that ranked the features according to their relevance to the class of samples; the other was called mRMR feature table that ranked the features according to the maximum relevance and minimum redundancy criteria. In the mRMR feature table, a feature with a smaller index implies that it is more important for the prediction of aptamer-target pairs. Such a list of ranked features was to be used in the following IFS procedure for the optimal feature set selection.

### IFS Result

**Figure 1:**
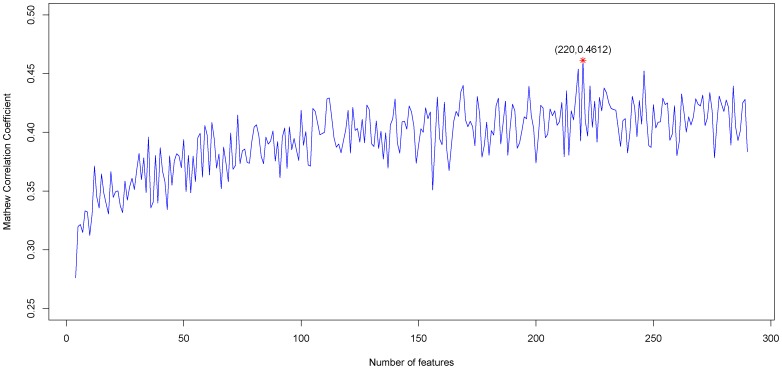
IFS curves showing the values of *MCC* against different number of features used based on the data in Additional File S5. When the first 220 features in the ranked feature list were used, *MCC* reached the maximum of 0.4612. These 220 features were considered as composing the optimal feature set for our prediction problem.

By adding the ranked features one by one, we built 290 individual predictors based on the 290 feature subsets for predicting aptamer-target pairs. We then tested the prediction performance of the 290 predictors and obtained the IFS results (see Additional File S5). Shown in Fig. 1 is the IFS curve plotted based on the data of Additional File S5. As we can see from the figure, the MCC reached the maximum value of 0.4612 when the first 220 features were used. Therefore, we regarded the 220 features as the optimal feature set for the prediction problem. Based on these 220 features, the prediction *sensitivity*, *specificity* and *accuracy* were 0.4879, 0.9218 and 0.8134, respectively (Table 1). Sn is the rate of aptamer-target pairs that are correctly predicted, while Sp is the rate for correctly predicted non-aptamer-target. For training dataset, Sn and Sp of our method is 0.4879 and 0.9218 respectively, due to the ratio between positives and negatives is 1∶3. However, for the random prediction, Sn and Sp should be 0.2500 and 0.7500, respectively. Therefore, our method increased the Sn and Sp by 0.2379 ( = 0.4879–0.2500) and 0.1718 ( = 0.9218–0.7500) respectively, which shows the effectiveness of our model. For these 220 features, please referred to the top 220 features listed in the Table mRMR in Additional file S4.

**Table 1:** Prediction performance on training dataset and testing dataset.

Dataset	Sn	Sp	Ac	MCC
Training dataset	0.4879	0.9218	0.8134	0.4612
Testing dataset	0.4828	0.8713	0.7741	0.3717

Sn: sensitivity.

Sp: specificity.

Ac: accuracy.

MCC: Matthews correlation coefficient.

### Prediction Performance on Independent Dataset

To assess the performance of our predictor, we applied our method on an independent dataset and achieved a *sensitivity* of 0.4828, *specificity* of 0. 8713, *accuracy* of 0. 7741 and *MCC* of 0. 3717 (Table 1). For independent testing dataset, Sn and Sp of our method is 0.4828 and 0.8713, respectively, also due to the ratio between positives and negatives is 1∶3. Our method increased the Sn and Sp by 0.2328 ( = 0. 4828-0.2500) and 0.1213 ( = 0.8713-0.7500) respectively, when compared with the random prediction on the dataset with the same composition.

### An Example of Correctly Predicted Aptamer-target Pair

**Figure 2:**
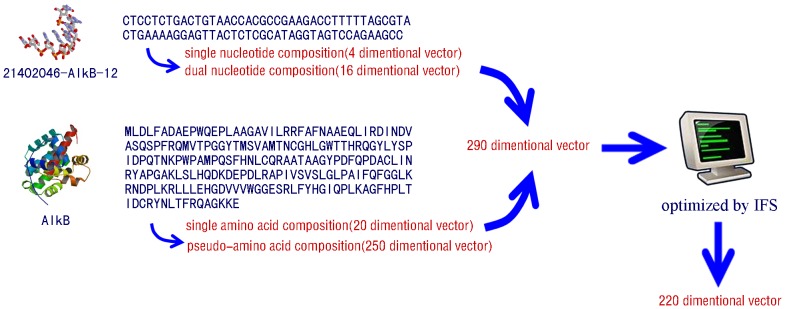
The feature extraction procedure of aptamer-target pair 21402046-AlkB-12: AlkB. Sequences of the aptamer and the target were shown, from which 290 features were extracted. Finally, 220 features were selected as the final optimal feature set from the 290 features by the IFS procedure, composing a 220-dimentional vector as input of the model.

Take the aptamer 21402046-AlkB-12 and its target AlkB as an example. The feature extraction procedure is illustrated in Fig. 2. The sequence of the aptamer 21402046-AlkB-12 contains 79 bases. The single nucleotide composition of A, T, C, G are 0.2658, 0.2405, 0.2152, 0.2785, respectively, which composed a 4-dimentional vector. Similarly, the dual nucleotide composition, i.e., the composition of AT, TT, TG, GG, etc., was computed respectively and they composed another 16-dimentional vector. In the sequence of the target AlkB, a 20-dimensional vector was computed from the 20 amino acid composition. The pseudo-amino acid composition of the target was also computed, composing another 250-dimensional vector, according to Ref [18]. Finally, the 21402046-AlkB-12:AlkB pair was represented as a 290 ( = 4+16+20+250) dimensions vector.

The 290-dimensional vector was then optimized by the incremental feature selection. Finally, a 220-dimensional vector was selected, in which the corresponding features were called the optimal features. The classifier constructed by the random forest with the optimal feature subset in the training set take the 220-dimensional feature of the 21402046-AlkB-12:AlkB pair as input and determined whether they interact or not as output.

Induced during an adaptive response, AlkB protein plays a role in the direct reversal of alkylation damage involved in DNA repairing. AlkB protein is recently detected by highly sensitive and selective aptamers selected in-house using CE-SELEX. Up to now, a few aptamers have been selected and were located differently on the AlkB protein [36], but had variability of affinity (dissociation constant, Kd). According Lock and Key Theory, the 3-dimensional structure of the aptamer such as size and shape of molecules explains the binding of an aptamer for the protein. Therefore, the structure of an aptamer’s target is one of factors considered by researchers. Ji Sun Choi et al. [37] explored the aptamers with a low nanomolar range binding affinity to demonstrated the binding sites of the aptamers for its targets appeared to be determined by the secondary structures. They predicted the secondary structures of the aptamer by computer program where a stem-loop secondary structure was investigated and the stem part (30-residue ssDNA sequence) was largely responsible for the binding. Nucleotide composition consists of the elemental information of aptamers and determines the intrinsic traits such as secondary structure. As a result, it is reasonable for this study to employ the nucleotide composition to depict aptamers.

### Analysis of the Optimal Features

**Figure 3:**
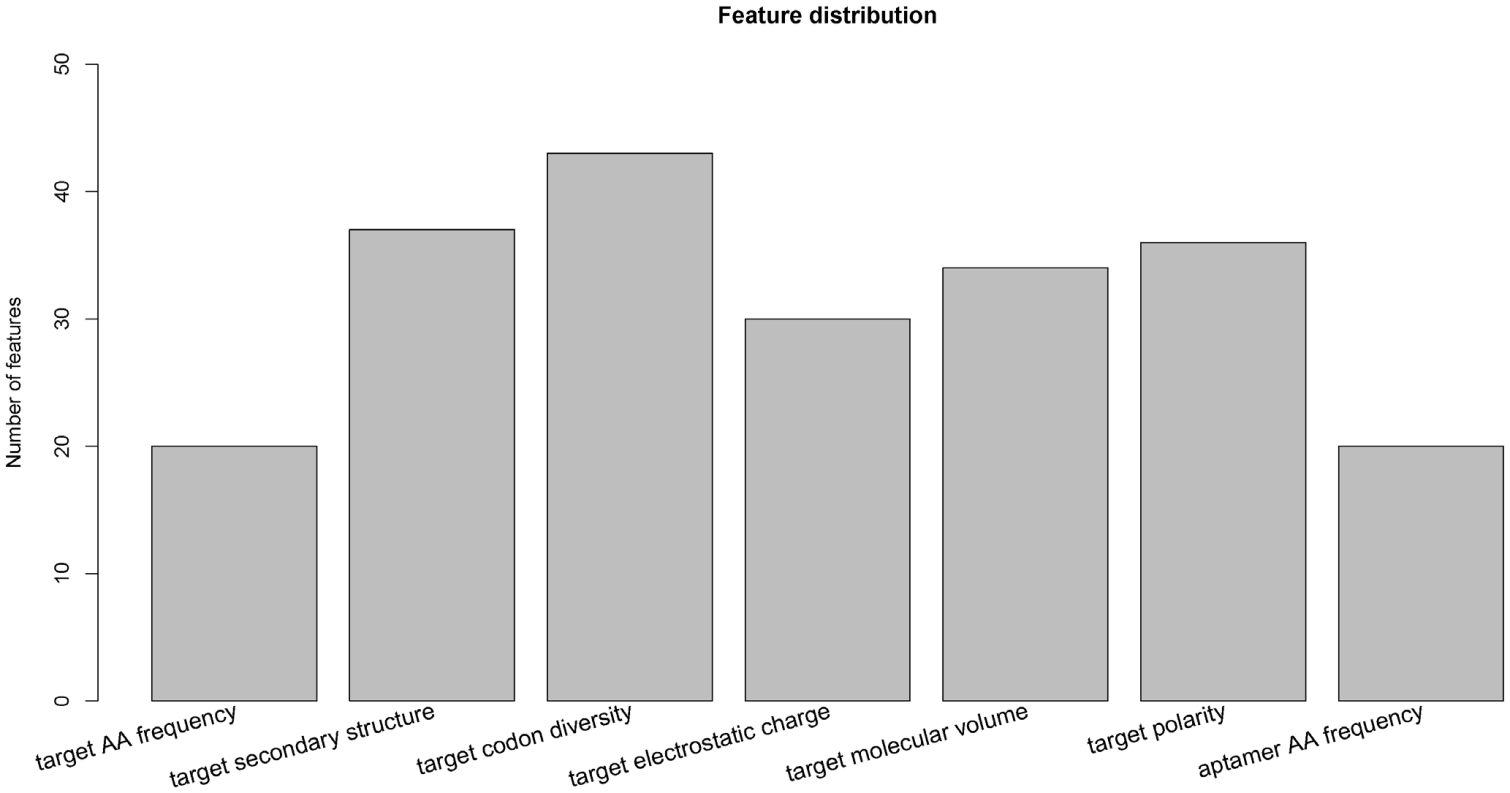
Histograms showing the distributions of the 220 optimal features.

The 220 optimal features derived from mRMR program can be categorized into 7 terms, namely, target frequency (amino acid composition), target secondary structure, target codon diversity, target electrostatic charge, target molecular volume, target polarity and aptamer frequency (the composition of nucleotide and dual-nucleotide) (Fig. 3).

The target codon diversity ranks the first making up approximately 20%. The codon diversity implies that the codon usage is an essential factor for aptamer-target interactions, because this trait to a large extent resides in the optimal features of interactions. This finding is consistent with the previous studies that proved codon usage play an important role in interaction related to proteins [38], [39], [40]. Probably, this is also stem from the reason that aptamers have to date been selected against a broad range of targets, including proteins (e.g. proteases, cell-surface receptors, kinases, cytokines, and cell-adhesion molecules), phospholipids, sugars, nucleic acids, as well as whole cells [41], [42], [43], [44]. Indeed, the applications of aptamers are widely exploited in the fields of diagnostics, therapeutics and medical imaging [45]. It suggests that our prediction of aptamer is based on a highly various scope of aptamer protein target and thus this prediction through mRMR program might be widely implemented into the design of aptamer.

Furthermore, the counts of features about conformational properties (secondary structure and molecular volume) as well as the trait of polarity nearly remain equal, with the number of about 35 in the optimal features. These traits take up a large part of the optimal features. This elucidates the importance of these traits and is consistent with the analyses of detecting subtle modifications with aptamer [46] and polarity of thrombin binding aptamer [47]. It has been proved that changes in the proteins can be detected by aptamers with specific activities [46] and the aptamer binding modified thrombin, which contains a 5′-5′ polarity inversion site, particularly has higher affinity and higher stability [47]. A subtle folding and harmonious charge density are beneficial for aptamers to recognize their targets effectively and selectively [48]. These conformational properties play crucial roles in interactions between small molecules and protein targets. For example, the size and position of functional groups of proteins are generally direct factors in interaction between molecules [49], [50], [51]. On the basis of Lock and Key Theory [52] which analyzed the effects of the size and shape of molecules in interactions, molecular volume is another main factor for strictly determining the affinity and specificity of interactions between aptamers and targets. Electrostatic charge of the targets is derived from the distribution and proportion of polar and charged amino acid residues. It facilitates forming short range interactions including salt-bridges and hydrogen-bonds. Little hydrophobicities in protein complexes and their interactions mainly benefit from the polar and charged residues [53], particularly in complexation when a small molecular binds to proteins [54]. Our results further affirm that the conformational and polar properties are important for identifying the pattern of interaction between aptamers and their targets.

As a result, our prediction of proper aptamer could be precise depending on the traits of conformation, polarity of proteins as well as electrostatic charge. Overall, our prediction through mRMR program is based on these targets’ propensities to interact with apatmers combining with certain pattern of amino acid and nucleotide composition selected in our prediction and thus it bestows us the ability to design several suitable aptamers to specifically recognize the given target protein.

## Conclusion

In this study, we developed a new method for predicting and analyzing aptamer-target pairs. Our method considered not only sequence information from aptamers but also traditional amino acid composition and pseudo amino acid composition from targets. By means of the feature selection algorithm, an optimal set of 220 features were selected. These features were regarded as the ones that contributed significantly to the prediction of aptamer-target pairs. With the 220 optimal features selected, our approach achieved an overall *accuracy* of 77.41% and 0.3717 *MCC* on an independent dataset. These selected features may shed some light on in-depth understanding of the mechanisms of interactions between aptamers and their targets, providing guidelines for designing novel and effective aptamers binding to certain interested targets.

## Supporting Information

File S1 **This file contains two sheets. The first one shows the positive samples of aptamer-target pairs.** The second one shows the negative samples of aptamer-target pairs.
(XLSX)

File S2 **The polarity, codon diversity, electrostatic charge, molecular volume and secondary structure of 20 amino acids.**
(XLSX)

File S3 **The training dataset consisting of the 580 positive samples and 1740 negative samples, the testing dataset consisting of the 145 positive samples and 435 negative samples, with each having the 290 feature components.**
(XLSX)

File S4 **This file contains two sheets.** The first one shows the MaxRel feature table, which ranked the features according to the relevance between features and class of the samples. The second one shows the mRMR feature table, which ranked the features according to the redundancy and relevance criteria.
(XLSX)

File S5 **The sensitivity (Sn), specificity (Sp), accuracy (Ac), Matthews’s correlation coefficient (MCC) of each run of IFS.**
(XLSX)
